# Exploring Salivary Metabolic Alterations in Type 2 Diabetes: Implications for Dental Caries and Potential Influences of HbA1c and Vitamin D Levels

**DOI:** 10.3390/metabo14070372

**Published:** 2024-06-30

**Authors:** Ashwaq Alkahtani, Martin Grootveld, Mohammed Bhogadia, Aylin Baysan

**Affiliations:** 1 Institute of Dentistry, Barts and The London School of Medicine and Dentistry, Queen Mary University of London, London E1 2AD, UK; a.s.s.alkahtani@qmul.ac.uk; 2 Leicester School of Pharmacy, De Montfort University, The Gateway, Leicester LE1 9BH, UK; mahb7784@gmail.com (M.B.); mgrootveld@dmu.ac.uk (M.G.)

**Keywords:** type 2 diabetes, dental caries, salivary metabolomics, biomarker signatures, ^1^H NMR analysis

## Abstract

Diabetes mellitus is considered to be the most common health issue affecting almost 1 in 11 adults globally. Oral health complications including xerostomia, periodontal disease, dental caries, and soft tissue lesions are prevalent among individuals with diabetes, and therefore an understanding of the potential association between salivary metabolites and dental caries progression would enable the early detection and prevention of this non-communicable disease. Therefore, the aim of this study was to compare salivary biomarkers between individuals with type 2 diabetes (T2DM) with those without this disorder (ND) using ^1^H NMR-based metabolomics strategies. The objectives were to identify T2DM-associated biomarker signatures and their potential impact on dental caries. In addition, HbA1c and vitamin D levels were also analysed for this purpose. Methods: Stimulated whole-mouth saliva (SWS) samples were collected from T2DM and ND (n = 30 in each case) participants randomly selected from a group of 128 participants recruited for this case–control study. All participants were advised to refrain from eating, drinking, and smoking for at least 1–2 h prior to sample collection. Following preparation, SWS supernatants underwent ^1^H NMR analysis at an operating frequency of 800 MHz, and the dataset acquired was analysed using a range of multivariate metabolomics techniques. Results: Metabolomics analysis of data acquired demonstrated that, together with up- and downregulated blood HbA1c and vitamin D levels, key salivary discriminators between these two classifications included lactate, taurine, creatinine, α-glucose, and formate to a lesser extent. The bacterial catabolites lactate and formate were both significantly upregulated in the T2DM group, and these have previously been implicated in the pathogenesis of dental caries. Significance analysis of metabolites (SAM)-facilitated AUROC analysis yielded an 83% accuracy for this distinction. Conclusion: In conclusion, this study highlights the significant differences in salivary metabolites between individuals with T2DM and healthy controls. Such differences appear to be related to the development and progression of dental caries in T2DM patients.

## 1. Introduction

Diabetes mellitus (DM) affects almost four million people in the UK, with over one-eighth of the population remaining undiagnosed, as specified by the Diabetes UK registration figures for 2020–2021 [1]. By 2025, it is estimated that five million people will have diabetes. Reported oral health complications associated with diabetes include dry mouth, periodontal problems, dental caries, and soft tissue lesions of the tongue with oral mucosa. Those co-morbidities, which are related to oral health, most especially involve periodontal tissues, and this association has previously been established [2,3]. Indeed, previous studies have demonstrated that selected caries factors such as reduced salivary flow rate and counts of *Mutans streptococci* can be related to the metabolic control of DM and may also influence the caries process. Moreover, throughout the last 20 years or so, further connections existing between oral diseases/periodontopathic bacteria and other systemic diseases in humans have been recognised [3]. These conditions include Parkinson’s disease [4], systemic lupus erythematosus [5], pancreatic and colorectal cancers [6], lung inflammation [7], and rheumatoid and osteoarthritis [8], in addition to type-2 diabetes [2,9]. Indeed, oral bacteria readily gain bloodstream entry, most especially in diseased states, and also during periods of mastication, flossing, and tooth-brushing episodes [3]. Interestingly, the hazards presented by periodontal diseases towards subjects afflicted with chronic diseases, e.g., diabetes [2], osteoporosis, and respiratory diseases, have only been quite recently realised [3]. Further relevant associations between oral and systemic diseases include those featuring heart disease and stroke, along with a greater risk status for preterm, low-birth-weight babies. Notwithstanding, the mechanistic factors involved in many of these co-morbidity relationships remain largely unknown, despite their epidemiological nature and establishment. For cardiovascular disease co-morbidity, one hypothesis has involved considerations of cross-reactions between bacterial and human heat shock proteins (HSPs) in periodontal infection, a phenomenon providing evidence for associations between these disorders and periodontal diseases [10]. Moreover, the development and progression of atherosclerosis in apoE-deficient mice is promoted by *P. gingivalis* infection [11]. Additionally, in a pilot study, Pardo et al. identified periodontal pathogens in both oral and cardiac specimens from patients enduring aortic valve replacement [12], and this again verified that oral infections may indeed serve as risk factors for systemic diseases. Therefore, the establishment of suitable controls for the management of oral conditions appears to be of much importance for the prevention and suppression of a range of systemic co-morbidities. 

Saliva represents a valuable biological fluid for investigating the molecular determinants of oral health and associated diseases, since it is relatively non-invasively collected, and contains a rich array of proteins, peptides, nucleic acids, and metabolites, which reflect the physiological and pathological status of the oral cavity. In addition, metabolic profiling of saliva has recently emerged as a powerful tool to identify novel biomarkers and gain insights into the biochemical pathways involved in oral diseases [13,14]. 

Therefore, comparative evaluations of the nature and levels of salivary biomarkers through metabolomics analysis between individuals with and without type 2 diabetes (T2DM and ND, respectively) may reveal potential biomarker patterns that enhance our understanding of the possible association between diabetes and dental caries, information which may be highly valuable to clinicians and researchers alike. In principle, these findings could aid clinicians regarding the early detection of dental caries, leading to effective preventative strategies. Ultimately, this would be expected to improve the quality of life for individuals with T2DM.

To date, we have presented results concerning the applications of high-field proton (^1^H) NMR spectroscopy to the detection and quantification of biomolecules present in a variety of complex biological fluids, and some examples from our work in the dental/oral health research area are available in [15,16,17]. High-resolution NMR analysis represents a bioanalytical technique that offers a plethora of advantages over a series of other analytical strategies, since, firstly, it allows the rapid, non-invasive, and simultaneous investigation of a very wide range of biomolecules present in biofluid samples (or further biological matrices), and, secondly, it is generally accepted that it requires only a limited amount of information regarding sample composition before analysis. Furthermore, sample preparation is very rapid and generally involves little or no modification to the liquid-state biofluid sample status, i.e., the addition of small volumes of deuterium oxide (^2^H_2_O) as a field frequency lock, and occasionally also a chemical shift reference and/or quantitative internal standard (e.g., sodium 4,4-dimethyl-4-silapentane-1-sulfonate (DSS), as employed here, together with options for the addition of a suitable buffering agent for PH maintenance, and perhaps also an added bactericidal agent such as sodium azide. Notably, the chemical shift (δ) values, coupling patterns, and coupling constants of signals visible in the ^1^H NMR profiles of complex, multicomponent biofluid samples furnish a high level of bioanalytical information concerning the molecular identity of both endogenous and exogenous agents detectable [16]. Furthermore, the envelope of broad overlapping resonances which is derived from any passively mobile biomacromolecules such as proteins present is regularly subdued via recommended spin echo pulse sequences, so that spectra arising therefrom comprise a large number of sharp, well-resolved signals arising from a wide range of low-molecular-mass (non-protein-bound) biomolecules, in addition to those from the molecularly mobile functions of macromolecules, such as the lipidic portions of lipoproteins [14,16]. With this approach, the sensitivity of the technique is ≤5 µmol/L at an operating frequency of 600 MHz [14]. 

Previously, our group has explored the abilities of both one- and two-dimensional high-field NMR technologies to characterise and monitor salivary biomolecules and also to provide valuable information regarding intra- and inter-subject variabilities of salivary metabolites concentrations, particularly organic acid anions, amino acids, and malodorous amines as bacterial catabolites [16]. Indeed, the investigation reported in ref. [16] has been described as being the very first ‘true’ untargeted metabolomics study conducted on human saliva samples [18].

Of especial interest to the current study, when used correctly, amalgamations of the multicomponent ^1^H NMR analysis technique with multivariate (MV) metabolomics strategies provide many benefits in clinical studies (e.g., diagnostic or prognostic), most notably because of the relatively restricted level of metabolic data obtained from the direct manual computer visual inspection of high-resolution spectra generated, which frequently contain data arising from >100 metabolites present per sample, a step which involves their quantification by specially trained staff, which is very costly, onerous and labour-intensive. Moreover, this metabolomics combination operates as a very powerful tool for discerning the biochemical fundament of human disease aetiology, together with the therapeutic or toxicological effects arising from the dose-dependent administration of drugs, or alternative xenobiotics [15,16]. Therefore, this approach would, in principle, ultimately contribute to optimal oral health outcomes and perhaps an improved quality of life status for individuals with T2DM. In this respect, the current study employed high-resolution nuclear magnetic resonance (NMR) spectroscopy as a primary metabolomics technique to characterise the salivary metabolic profiles of study participants, and subsequently identify any metabolic signatures or patterns putatively associated with both T2DM and dental caries. The hypothesis was that modifications in salivary metabolites and their concentrations, which are mediated by T2DM, may influence the development, progression, and severity of dental caries. 

Unfortunately, previous studies focused on explorations of the metabolic profiles of patients with T2DM and its association with oral health conditions are somewhat limited, and therefore this investigation aimed to compare the status of salivary metabolites in a case-controlled study involving individuals with T2DM and corresponding age-matched healthy controls. In addition, whole-blood HbA1c and vitamin D levels were analysed in these recruited participants in order to assess their relationship to any potential disease-mediated modifications observed in ^1^H NMR-determined salivary metabolite concentrations.

## 2. Materials and Methods

### 2.1. Sample Populations 

This investigation was part of a case–control study (Ethical Committee Code: 20/NW/0007). All participants provided written informed consent prior to their involvement in the study. It consisted of two groups of participants: those with type 2 diabetes (T2DM/n = 30) and non-diabetic (ND/n = 30) control individuals. A total of 60 whole stimulated saliva (SWS) samples were collected from randomly selected sub-groups of 30 participants per group, in accordance with the specified exclusion and inclusion criteria (available in Supplementary Table S1). The mean ± SD ages of the ND and T2DM groups were 54.33 ± 12.77 and 65.00 ± 10.87 years, respectively (13 males/17 females and 18 males/12 females, respectively). The percentages of races and ethnic groups for the entire n = 60 participants recruited to the study were 21.67% Asian British; 28.33% White/Caucasian; 21.67% Black/African British; and 28.33% other. The full impact of sociodemographic variables and T2D, and oral health behaviours, together with dietary practices, on oral health outcomes in this cross-sectional study was reported in ref. [19]. Indeed, this report found that being of the male gender, Asian British, retired through disability, a polypharmacy user, and with removable prostheses were all significant factors for T2DM risk. Overall, this was an observational study involving a single visit to the clinic. There were no study dropouts. The screening of T2DM and ND participants for diabetes or non-diabetes is described below.

During the screening process for study participants, individuals who had not previously been diagnosed with any type of diabetes were recruited for the ND group, while those who had been officially diagnosed with T2DM were assigned to the T2DM group. Subsequently, during the assessment appointment, an HbA1c test was performed using the Quo-Test^®^ A1c point-of-care testing (POCT) analyser, which operates with a finger-prick blood sample. The threshold HbA1c value used to confirm individuals as non-diabetic was set at ≤48 mmol/mol (6.5%). Only one of the 27 participants in the ND group had an HbA1c value >42 mmol/mol (6.0%), the threshold level for pre-diabetes, but this was only a very marginally higher value (42.3 mmol/mol).

Mean ± SD values for HbA1c concentrations in the ND and T2DM groups were 35.70 ± 5.94 and 54.15 ± 13.03 mmol/mol, respectively (Table 1), and these correspond to percentage values of 4.83 ± 0.80 and 7.33 ± 1.76%, respectively. As expected, a two-sample *t*-test performed on these raw data confirmed that there was a very highly significant difference between the mean whole-blood HbA1c levels of these two groups (*p* = 1.11 × 10^−8^), with the T2DM one being much greater.

Also listed in Table 1 are further clinical characteristics for the participants recruited to the study, including the International Caries Detection and Assessment System (ICDAS) index, which was significantly higher in the T2D group, as we might expect, and the percentage distribution of root caries lesions, which was also much higher in this group. However, the extreme risk of dental caries, also determined as a percentage, was approximately three-fold greater in the ND control group. 

#### 2.1.1. Determination of Participant Whole-Blood Vitamin D (25-Hydroxyvitamin D) Concentrations for the ND and T2DM Groups 

This study utilised the vitamin D dry blood spot test kit (Blood Spot Test for Vitamin D Public Service, Black Country, West Midlands, NHS, UK) to assess vitamin D levels in each of the ND and T2DM group participants, as one of its active metabolites, 25-hydroxyvitamin D. This test kit was specifically designed for this purpose and enabled the collection of blood samples on a filter paper, which was then used for the measurement of 25-hydroxyvitamin D levels. The method offered a convenient and reliable means of assessing vitamin D status and was found to be highly valuable for this research investigation which requires quite large-scale sample cohort collections. For these concentrations, the following reference ranges were used: extremely deficient 15 nmol/L; deficient 15–30 nmol/L; insufficient 30.1–50 nmol/L; adequate 50.1–220 nmol/L; high to toxic 220.1–500 nmol/L; and toxic >500 nmol/L.

Whole-blood mean ± SD 25-hydroxyvitamin D levels for the ND and T2DM group participants were found to be 72.08 ± 32.51 and 69.26 ± 37.20 nmol/L, respectively. A two-sample two-tailed *t*-test showed that there were no significant differences between these values (*p* = 0.783). Nor were there any differences between sample variances (F variance ratio statistic). No significant correlation whatsoever was found between the whole-blood HbA1c and 25-hydroxyvitamin D levels for all study participants (r = 0.036). 

#### 2.1.2. Sample Size Power Calculation for the Study 

In view of the unavailability or very limited number of bucketed ^1^H NMR data prior to commencing the current study, the sample size chosen for this investigation was estimated in a univariate context via the consideration of a highly significant (*p* < 0.001) correlation of dental caries group-upregulated salivary formate concentrations with patient dental caries experience, with the latter reflected by the number of decayed and filled surfaces (dfs), as documented in ref. [20]; formate was found to represent a very important biomarker in this study. Hence, the sample size per group was estimated using 90% confidence interval (CI) bounds with 10 and 20% alpha- and beta-error values, respectively, using the r (Pearson’s correlation coefficient) parameter determined for the relationship between dfs values and salivary formate concentrations (+0.487). The expression employed for sample size estimation was n = ([Z_α_ + Z_β_]/C)^2^ + 3, where Z_α_ = 1.6449 and Z_β_ = 0.8416 normal deviate values, and C = 0.50 × n[(1 + r)/(1 − r)]. From this formula, the sample size estimated per group was n = 30. However, funding available and economic factors provided further constraints to sample size selection for each group evaluated.

### 2.2. Sample Collection

All participants were advised to refrain from eating, drinking, and smoking for at least 1–2 h. prior to saliva sample collection. To collect stimulated whole saliva (SWS), participants were instructed to chew a piece of standard chewing wax for one minute and then expectorate the first component. Subsequently, they were directed to spit their saliva into a labelled sterile plastic vial every 30 s until an estimated 5–10 mL of this biofluid was obtained. To maintain sample stability and minimise bacterial degradation of salivary proteins and other biomolecules, all saliva tubes were placed in diagnostic specimen containers with dry ice (DANIELS^®^, Dorset, UK) until the end of the assessment appointment. Samples were then transferred and stored at −80 °C until all samples were collected and prepared for ^1^H NMR analysis.

### 2.3. Sample Preparation and ^1^H NMR Analysis 

#### 2.3.1. Sample Preparation

The frozen saliva samples were thawed on ice, and 1.0–1.5 mL aliquots of each were aliquoted into Eppendorf tubes and centrifuged at 10,000 rpm for 30 min. to remove particulate matter, i.e., cells and debris. For acquisition, a mixture of 450 µL of each salivary supernatant with 50 μL of an NMR-based buffering system (containing 1.50 mol/L KH_2_PO_4_, 2.00 mmol/L sodium azide, and 5.80 mmol/L DSS (Sigma-Aldrich, Milwaukee, WI, USA)) in deuterium oxide (D_2_O, Cambridge Isotope Laboratories Inc., Boston, MA, USA), PH 7.40. DSS served as an internal chemical shift reference standard (δ = 0.00 ppm), and D_2_O acted as a field frequency lock. The mixture was then vortexed for 30 s and again centrifuged, but for this step at 12,000 rpm for 5 min. Subsequently, 500 µL aliquots of the supernatant was transferred to 5 mm diameter NMR tubes for ^1^H NMR analysis. 

#### 2.3.2. Acquisition of ^1^H NMR Spectra 

^1^H NMR spectra were acquired on a Bruker Avance HD III spectrometer operating at a proton frequency of 800 MHz. Samples were maintained at 4 °C prior to analysis. Samples were analysed at 25 °C following a 5 min. period for temperature equilibration. One dimensional ^1^H NMR spectra were acquired using a nuclear Overhauser effect spectroscopy (NOESY) pulse sequence coupled with water presaturation, a relaxation delay of 4 s, and an acquisition time of 2.73 s, with 128 transients collected using 65,536 data points, following 8 dummy scans; the spectral width was 15 ppm (−2.8 to 12.2 ppm). 

#### 2.3.3. Preprocessing of ^1^H NMR Profile Data 

The experimental strategy involved the addition of all spectra acquired into one common file, in which the ‘intelligent bucketing algorithm’ examined all spectra simultaneously and focused on the ‘bucket limits’ of commonly observed resonance intensity areas. The ISB approach employed ensured that all bucket edges involved did not coincide with ^1^H NMR resonance maxima, and this process therefore prevented the splitting of signals across separate integral regions; a 0.04 ppm bucket width with a 50% looseness factor was employed. This strategy generated one global table of intelligently selected bucket (ISB) intensities, which was then imported into MS Excel for further manipulations. These computations were executed using the ACD/Labs 1D NMR Manager software package version 2022 for Microsoft Windows (Advanced Chemistry Development, Inc. (ACD/Labs), Toronto, ON, Canada). Resonance assignments for WMS samples were made by a routine consideration of chemical shift values, coupling patterns, and coupling constants, and also with a reference to sources from the literature, including ref. [18], Grootveld et al. [14,16], and the Human Metabolome Database [21]. 

Following the application of this preprocessing strategy, n = 3 of the ND group failed to provide acceptable good quality ^1^H NMR profiles, and therefore these samples were removed from the complete dataset prior to the application of further spectral editing as described below, followed by the application of MV data analysis.

Primarily, spectral editing was conducted to remove the broad water signal (δ = 4.80 ppm), along with that of the ^1^H NMR resonances of all potentially interfering exogenous agents released by the chewing wax product employed to stimulate saliva generation. These chewing wax material signals and their spectral regions were those of (1) polyols (D-glucitol/D-xylitol/D-mannitol), i.e., -CHOH/CH_2_OH resonances located in the δ = 3.57–3.90 ppm range; (2) stearate and glycerol fatty acid esters (GFAEs) δ = 0.85–0.89 (terminal-CH_3_), 1.26–1.29 (bulk chain -(CH_2_)_n_-), 1.50–1.56 (β-CH_2_), and 2.30–2.34 ppm (α-CH_2_); GFAE glycerol backbone 4.27–4.32/4.33–4.38 (-CH_2_OH) and 5.27–5.34 ppm (-CHOH); and enzactin δ = 2.13–2.15 ppm (2 × -CH_3_CO-), 2.34–2.36 (1 × CH_3_CO-), 4.07–4.11 (-CH-O-), and 4.22–4.25 ppm (2 × -CH_2_-O-). Moreover, in virtually all spectra acquired, there was a very highly intense signal (an apparent quartet) located at approximately 2.10 ppm, and therefore the 2.08–2.125 ppm region was removed from all salivary supernatant spectra acquired. Additionally, since the DSS internal standard employed was undeuterated, buckets for its characteristic resonances located within the δ = 0.60–0.65, 1.72–1.78, and 2.88–2.93 ppm ranges were also removed from these spectra prior to performing multivariate (MV) metabolomics analysis. Resonance assignments for these exogenous agents were made via reference to a wide range of sources from the literature, including ref. [21]. 

Subsequently, for the purpose of MV data analysis, all remaining endogenous salivary metabolite ISB intensities were constant sum-normalised (CSN) in order to overcome any issues arising from variable salivary flow rates amongst all participants. These datasets were then generalised logarithmically (glog)-transformed and Pareto-scaled prior to performing MV data analysis. Thereafter, unsupervised principal component analysis (PCA) was primarily employed in order to screen for any outliers; one was detectable in plots of PCs 2–4 vs. 1, and therefore this sample, from the T2DM group, was removed prior to proceeding with further MV analysis using either partial least squares discriminatory analysis (PLS-DA), orthogonal partial least squares discriminatory analysis (OPLS-DA), and MV area under the receiver operating curve (AUROC) analyses. Therefore, the final dataset subjected to both univariate and MV data analysis consisted of n = 27 ND and n = 29 T2DM participants. 

### 2.4. MV Metabolomics Analysis

#### 2.4.1. PLS-DA, OPLS-DA, and AUROC Biomarker Analyses

All MV data analysis was performed using MetaboAnalyst 6.0 software (the University of Alberta and National Research Council, National Institute for Nanotechnology, (NINT), Edmonton, AB, Canada). PLS-DA is a supervised analysis approach that employs MV regression techniques to excerpt information that may be valuable for predicting disease group classifications from a linear combination of the original biomolecule resonance intensity/concentration variables, which, as in PCA, are linearly correlated within each component isolated; however, all components included remain orthogonal. Similarly, OPLS-DA also serves as a powerful technique for dimensionality reduction and the seeking of metabolites (or equivalent ^1^H NMR profile buckets) which navigate distinctions between two disease groups for comparison. Indeed, it can be employed either with or instead of PLS-DA in view of its abilities to distinguish between group, classification-specific dataset variations from those irrelevant to this source of variation, i.e., the ‘between-participant-within-group’ source in this case. Variable importance parameters (VIPs) were reported for both these types of MV discriminatory analysis strategies. 

Distinctions found between the two groups with the above PLS-DA and OPLS-DA strategies were cross-validated using permutation tests with 2000 permutations. This strategy was employed in view of the knowledge that PLS-DA models using the Q^2^ statistic for cross-validation purposes (using five-fold resampling in this case) are less powerful for the detection of small differences between two classification discriminations than those in models employing the number of misclassifications (NMC) or AUROC as diagnostic criteria [22]. The five-fold resampling method applied involved the randomised partition of the dataset into five portions, four of which were used to construct models, without contributions from cases within the fifth data subset portion, and which were employed for evaluation purposes only.

AUROC analysis was also applied to the constant sum-normalised, glog-transformed, and Pareto-scaled dataset using the Biomarker Analysis module of the Metaboanalyst 6.0 software suite. Both classical univariate and more appropriate MV options available were applied. The MV form of this analysis included cross-validation approaches using support vector machine (SVM) techniques. ROC curves were produced via Monte-Carlo cross-validation (MCCV) employing a balanced sub-sampling approach. For each MCCV performed, two-thirds of the total number of saliva samples (participants) were utilised to construct models for determining the importance of each biomarker/NMR bucket feature variables (i.e., the ‘training’ set), and the top 2–60 important variables were then employed for building models with selected numbers of these, which were then validated on the one-third of samples left out (classically known as the ‘test’ set). This process was then repeated many times in order to compute 95% confidence intervals (CIs) and assess the overall importance of each model constructed. The classification and feature ranking methods employed were the linear SVM and SVM built-in strategies, respectively.

Using the variable ‘hold-out’ function of the software utilised, the utility of the ^1^H NMR-only dataset was evaluated in this manner, i.e., the blood biomarkers HbA1c and 25-hydroxyvitamin D were removed, and the AUROC analysis repeated, again using the linear and built-in SVM approaches for classification and feature ranking assessments, respectively. For this analysis model, both CSN and product quotient (PQN) row-wise normalisation methods were applied, the latter involving the use of a mean ND control spectrum as a reference normalisation standard. 

#### 2.4.2. Further Assessment of AUROC Feature Selection Pathways

In order to further assess and validate the predictive value of the biomarker metabolite variables selected using analysis with the AUROC technique, and also correct the key ones selected by this analysis for the false discovery rate (FDR), the significance analysis of metabolites (SAM) [23], and the empirical Bayesian analysis of metabolites (EBAM) [24] strategies were selected. Indeed, the SAM approach is designed to solve FDR issues experienced with the operation of multiple tests on multidimensional datasets. For this purpose, it primarily provides significance scores to all predictor variables, which are based on its modification when expressed relative to the ‘repeated’ (‘between-participant’) variable standard deviation. Subsequently, this technique then selects variables with scores that are higher than an ‘adjustable’ threshold value, and then compares such relative differences to the null distribution estimated by random permutations of the disease classification group labels (i.e., T2DM or ND labels in this case). For all threshold values employed, a percentage of permutation set variables will be found to be significant purely by chance, and in this study, this proportion was employed to compute the FDR value. Correspondingly, the EBAM algorithm, which represents a modification of the SAM technique, was also applied, with the difference being that for this technique, a modified t statistic was utilised to compute score values. Default delta values of 0.30 and 0.90 were employed for SAM and EBAM analyses, respectively. 

Following the application of this FDR-corrective procedure, AUROC analysis was then repeated on a dataset limited to the seven predictor variables which were found to be significant via application of the FDR-corrected SAM model. 

## 3. Results

### 3.1. ^1^H NMR Analysis of SWS Supernatant Samples and Their Resonance Assignments

**Figure 1:**
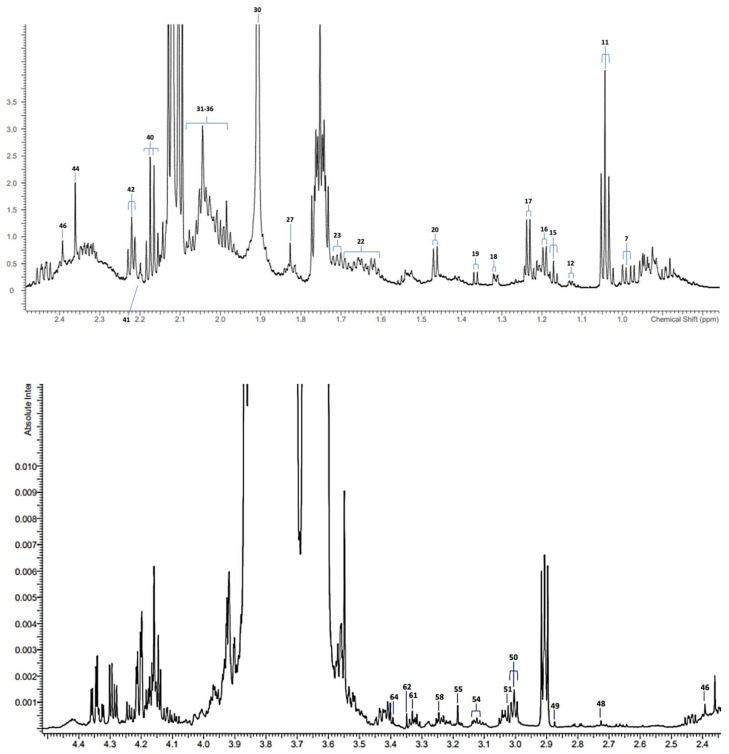

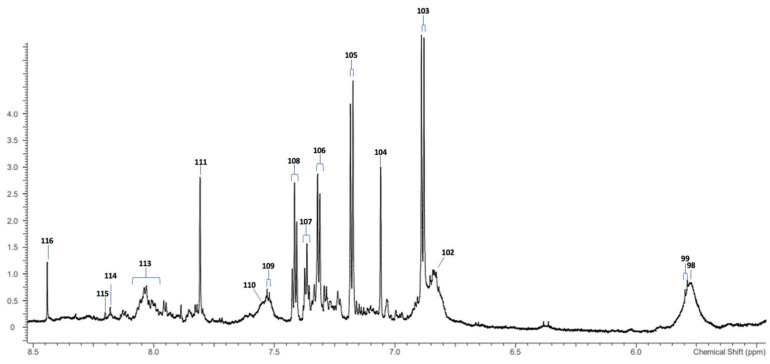
Partial ^1^H NMR profiles of a saliva sample collected from an ND group participant. Diagrams show the 0.80–2.50 (**top**), 2.35–4.50 (**middle**), and 5.50−8.50 ppm regions (**bottom**) of spectra acquired. A typical spectrum is shown. Resonances specified as uncoded in Table 2 were visible in general, but not in the spectral profiles shown in the figure. Abbreviations: assignment codes correspond to those listed in Table 2.

A typical high-field (800 MHz) ^1^H NMR spectral profile of a SWS supernatant sample from the ND group of study participants is shown in Figure 1, along with corresponding assignment codes for a total of 40 resonances detectable. Table 2 shows the salivary metabolite resonance assignments, along with their respective chemical shift values and coupling patterns. The spectral regions of agents released from the chewing wax stimulatory matrix employed, which are listed above in Section 2.3, were primarily removed since they clearly acted as at least partial bioanalytical interferents. 

### 3.2. MV Metabolomics Analysis of ^1^H NMR Spectral Datasets

#### 3.2.1. Application of PCA, PLS-DA, and OPLS-DA Techniques

**Figure 2:**
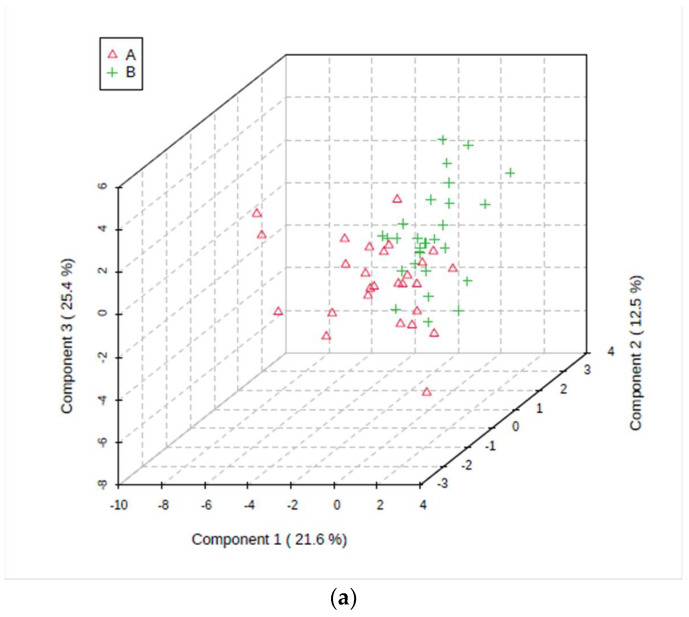

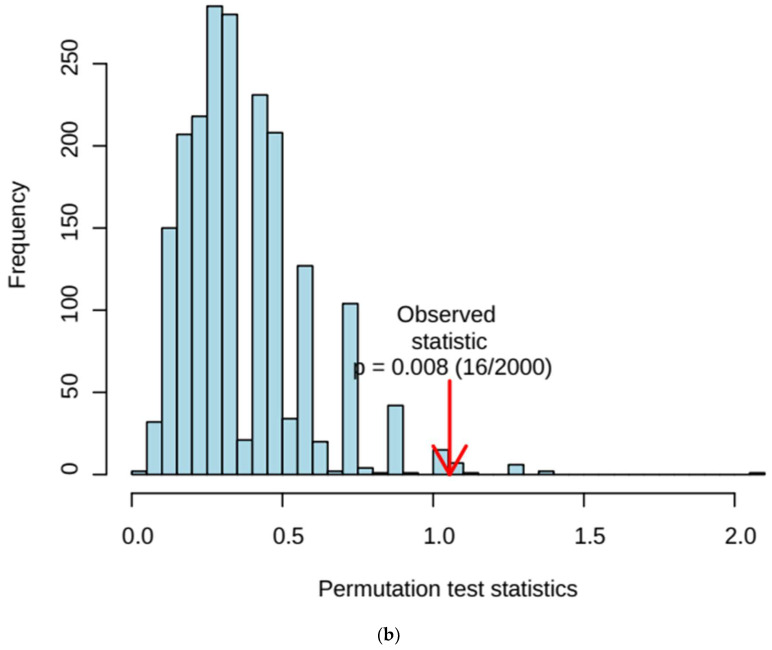
(**a**) A three-dimensional (3D) PLS-DA scores plot displaying some level of metabolite pattern distinction between the ND and T2DM groups. The red triangles indicate saliva samples obtained from ND participants (A), and the green crosses indicate those collected from the T2DM participant group (B). (**b**) Validation of the PLS-DA model applied by the application of 2000 permutation tests, which were based on separation distance (*p* = 0.008).

**Figure 3:**
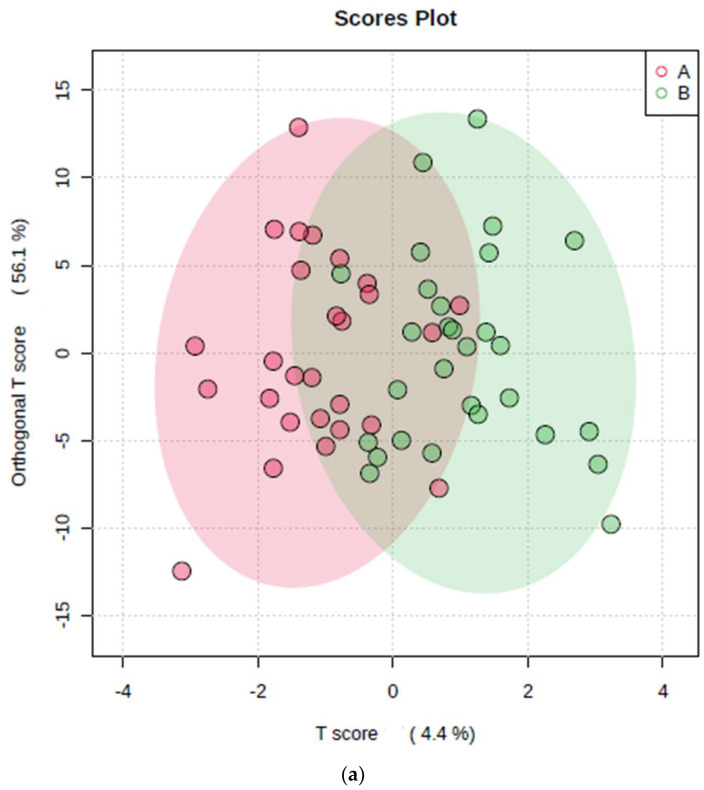

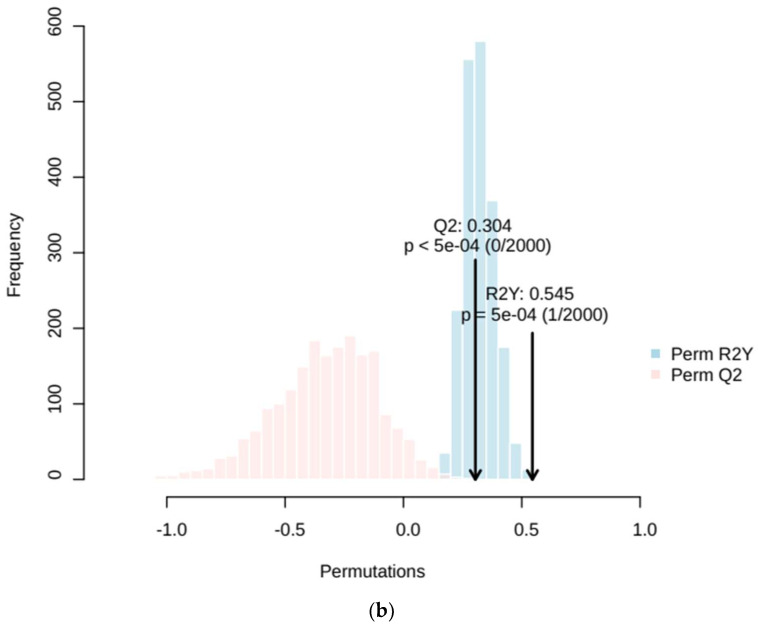
(**a**) Orthogonal partial least squares discriminant analysis (OPLS-DA) scores plot of high-resolution ^1^H NMR data derived from saliva samples donated by the T2DM and ND groups. The red sample data points indicate saliva samples obtained from the ND group, whereas green points indicate those obtained from the T2DM group. (**b**) A permutation analysis of the OPLS-DA model was established, which also shows the observed and cross-validated R^2^Y and Q^2^ coefficient values (*p* = 5.00 × 10^−4^ for Q^2^). An MV AUROC analysis.

As a first unsupervised approach, PCA was applied to all available samples from both groups to provide an overview of the variation in the complete dataset. Subsequently, the supervised PLS-DA and OPLS-DA techniques were applied. Overall, from 2D PCA score plots, it appeared that with the exception of one or two minor transgressions, and the removal of one outlying sample as described in Section 2.3, no further unacceptable major outlier samples were detectable in these plots. These potential further outliers, which were all T2DM participant samples, were also visible in the 3D PLS-DA scores plot attained on bucketed 1D NOESY spectra colour-coded by participant status, i.e., component 3 vs. component 2 vs. component 1 sample score values of the dataset, which is shown in Figure 2. However, the corresponding 2D OPLS-DA scores plot shown in Figure 3 revealed no major outlying data points. 

Permutation of sample group labels of the PLS-DA dataset was performed in order to validate the model and determine its ability to predict the disease classification (i.e., ND vs. T2DM groups). This testing system yielded a *p* value of 0.008 (Figure 2b), and this confirmed the valuable predictive capacity of this model. Similarly, a cross-validation permutation testing of the OPLS-DA model gave *p* values of <5.0 × 10^−4^ for both its Q^2^ and R^2^Y coefficient values (Figure 3b). 

Table 3 lists the variable importance parameter (VIP) values of metabolites and their signatory insights from the application of the PLS-DA MV analysis model. The most important metabolites for differentiating between the two groups according to this model were salivary taurine and creatinine, a superimposed salivary metabolite signal located at 3.10–3.15 ppm, blood HbA1c, salivary lactate, and blood 25-hydroxyvitamin D. Taurine, creatinine, lactate, and HbA1c, along with the unassigned metabolite, were upregulated in T2DM (as expected for HbA1c), whereas 25-hydroxyvitamin D was upregulated in the ND group (as expected). α-Glucose, glyc A glycoprotein, urea, phenylalanine, methanol, and formate were also identified as contributing to the differentiation between the two groups; however, their VIP values ranged from 0.90 (formate) to 1.17 (urea and GlycA glycoprotein), and therefore they were considered to be less important than those noted above. 

Table 4 shows the results obtained from the application of the OPLS-DA technique to this dataset, which identified HbA1c, vitamin D, lactate, GlycA glycoprotein, lysine, proline, and taurine as the most important metabolites for differentiating between the two groups. HbA1c and vitamin D were upregulated and downregulated, respectively, in the T2DM group, as we might expect, whilst GlycA glycoprotein, lysine, and proline were upregulated in the ND group. Lactate and the β-amino acid taurine were also significantly upregulated in the T2DM sampling group. The other metabolites identified by the OPLS-DA approach, such as creatinine, an unassigned metabolite, tyrosine, and histidine, also contributed to the differentiation between these two groups, although less so than the above metabolite variables, with VIP values ranging from 1.01 to 1.19. 

The OPLS-DA model applied to the dataset yielded a Q^2^ value of 0.30, although the permutation test conducted for it was very highly significant (*p* = 5.00 × 10^−4^). A 2D scores plot and associated cross-validation details for this model are shown in Figure 3. 

**Figure 4:**
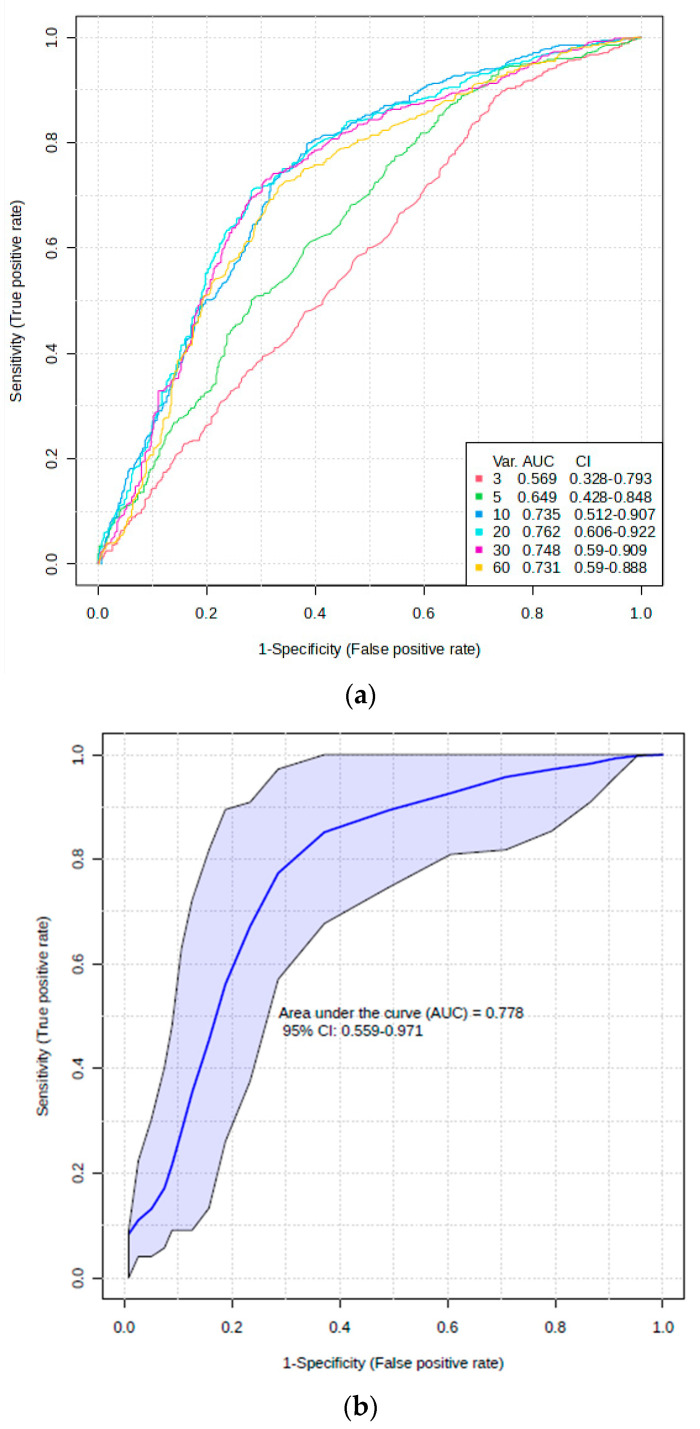
A multivariate AUROC analysis. (**a**) The model receiver operating characteristic (ROC) area under curves (AUROCs) for 6 PLS-DA classification models. The key shows the number of features for each model, with their respective AUROC values and associated 95% confidence interval (CI) values. Models 1, 2, 3, 4, 5, and 6 had 3, 5, 10, 20, 30, and 60 predictor variables incorporated, respectively. From their AUROC values, the best model was obtained with 20 predictor variables/metabolites (model 4). (**b**) The receiver operating characteristic (ROC) area under the curve (AUC) plot for PLS-DA classification model number 3, with 10 feature variables. The 95% confidence interval ellipse (CIE) for this AUROC model is also shown.

Receiver operating characteristic (ROC) curves were also plotted for six models simultaneously (Figure 4a), each with a different number of variables incorporated (3 to 60). The AUROC values for all six models are visualised in different colours in Figure 4a, and these are provided in the key, along with their 95% confidence interval (CI) ranges. As noted from the AUROC values, the best models were obtained with a minimum of 10 and a maximum of 20 variables incorporated (AUROC values of 0.735 and 0.762, respectively). Figure 4b shows the ROC curve and AUROC value for model number 3 with 10 variables and its associated 95% CIs. Models 3–6, i.e., those with 10–60 predictor variables, were all statistically significant, since their lower 95% CI bounds were >0.50.

**Figure 5:**
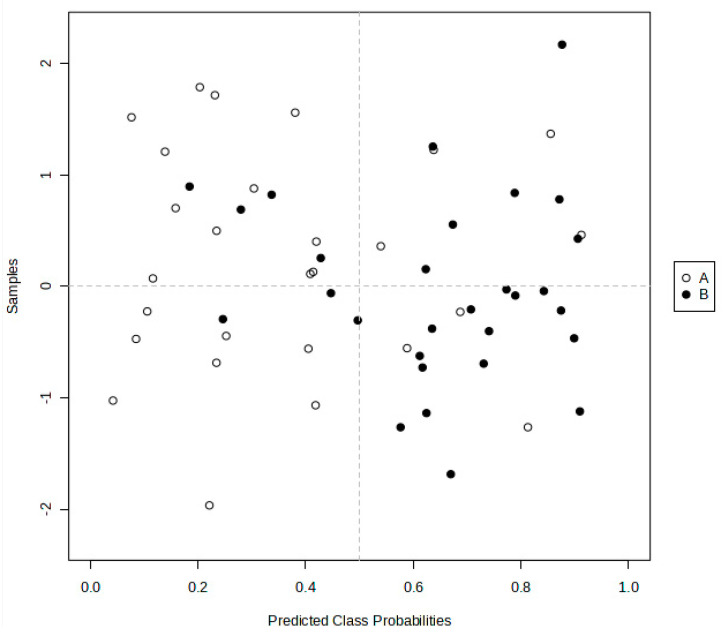
Predicted class probabilities (average of the cross-validation) for each sample using the best classifier model (based on the AUROC values obtained). The classification boundary was set to PLS-DA classification model number 4, with 20 metabolite variables. The dotted vertical line represents the distinction boundary. The hollow dots depict class A (ND), while the solid dots depict class B (T2DM). This analysis demonstrates that 21/27 ND and 22/29 T2DM participants were correctly classified.

Figure 5 shows the predicted class probabilities (average of the cross-validation) for each sample using the best classifier (based on its AUROC value). Since a balanced sub-sampling approach was used for model training, the classification boundary is always at the centre of the plot (*x* = 0.50, the dotted line). Therefore, according to this model, samples with probabilities of less than 0.5 were classified as group A (ND), whilst samples with probabilities greater than 0.5 were classified as group B (T2DM). As is evident from this figure, seven T2DM samples were misclassified as the ND classification, whereas six ND samples were misclassified as T2DM. Hence, the true positive rate (TPR) for group A was 21/27, whereas that for group B was 22/29.

Table 5 provides a summary of the 17 most significant variables obtained by AUROC analysis only in this study, with details of NMR chemical shifts, metabolite identities, and their regulatory status, together with corresponding univariate AUROC values for the dataset. The predictive accuracy for the best AUROC model with 20 predictor variables was 71.4%. 

#### 3.2.2. AUROC Analysis of ^1^H NMR ISB Variables Alone

Cross-validated AUROC analysis was also performed on a ^1^H NMR dataset only in order to evaluate the performance of models constructed therefrom. This analysis employed either constant sum or product quotient normalisation approaches (the latter using a mean ^1^H NMR spectral profile for the ND group as a reference), together with glog transformation and Pareto-scaling. The results acquired demonstrated that the best models for the CSN dataset were those with 10 or 20 predictor variable features (AUROC 0.707 (0.50–0.91) and 0.722 (0.52–0.86), respectively, with 95% CIs in brackets). For the 20-variable model, 21/27 ND and 24/29 T2DM were correctly classified, and its overall accuracy was 67.4%. 

Similarly, those for the PQN dataset had 5 or 10 important variable features (AUROC 0.72 (0.51–0.97) and 0.76 (0.51–0.99), respectively). The 10-variable model yielded 21/27 ND and 25/29 T2DM participants correctly classified, and the overall accuracy of the model was 69.6%. For both these MV analysis approaches, significant ^1^H NMR-based only variables were found to be formate (↑) > lactate (↑) > Glyc A/N-acetylsugars (↓) > histidine (↓) > phenylalanine (↑) > α-glucose (↓) for the CSN format, and formate (↑) > lactate (↑) > Glyc A/N-acetylsugars (↓) > phenylalanine, and (↑) > α-glucose (↓) for the PQN version. 

Therefore, in view of the width of the 95% CIs found for these analyses, and their overall accuracies, it is clear that the dataset also containing the non-^1^H NMR ISB whole-blood variables HbA1c and 25-hydroxyvitamin D provided an improved model when analysing the CSN dataset. 

#### 3.2.3. SAM- and EBAM-Supported AUROC Analysis: Significance Level Adjustments for FDR

**Figure 6:**
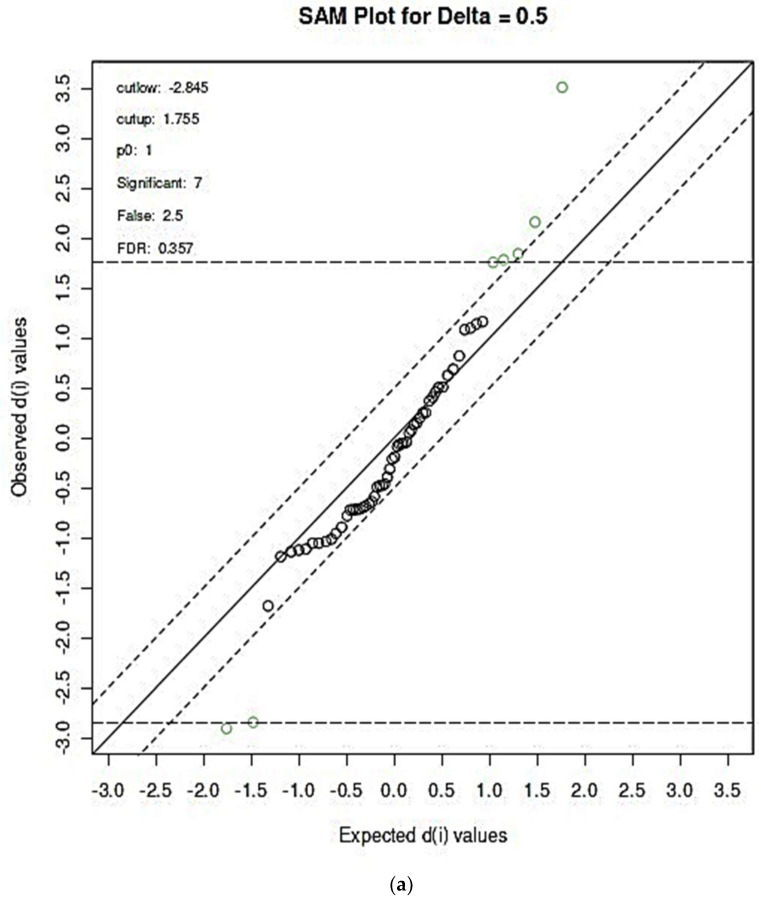

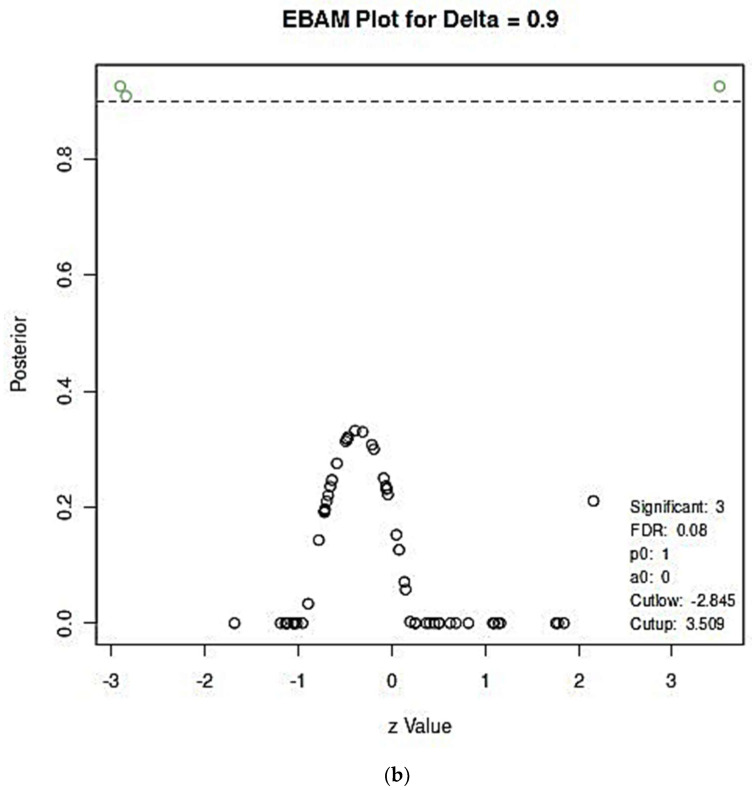

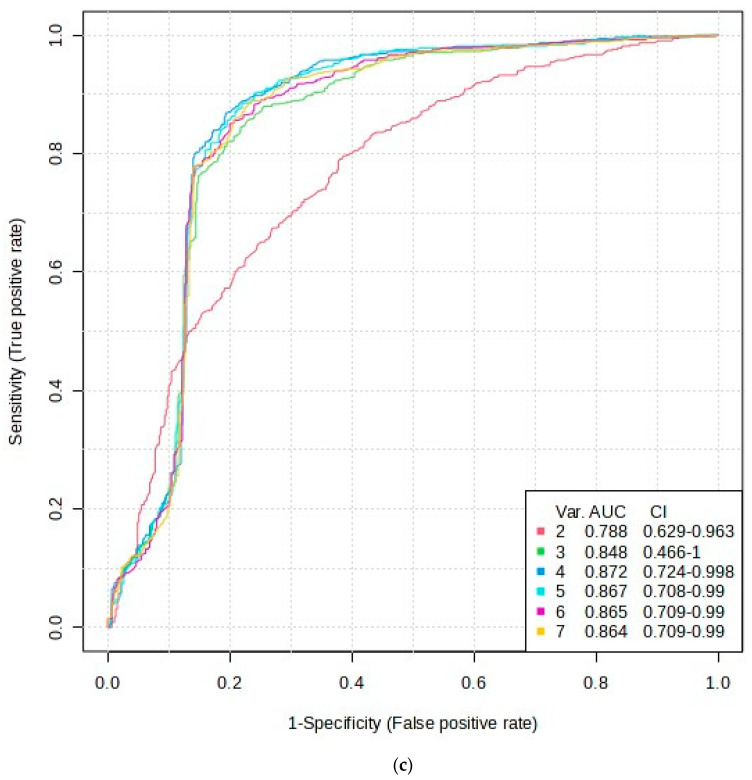
Significant predictor variable features identified by the application of the (**a**) SAM and (**b**) EBAM techniques to the dataset. Delta values for these analyses were set to their default values of 0.50 and 0.90, respectively. The green circles show the variable features that exceeded the specified thresholds. (**c**) ROC curve analysis of the seven significant biomarker variable datasets extracted from the FDR-corrected SAM analysis shown in (**a**).

Further validation of the AUROC analysis approach was performed using the SAM and EBAM techniques, which corrected the statistical significance of biomarkers detectable through the application of false discovery rate (FDR) adjustments. Results from these analyses are shown in Figure 6a and Figure 6b, respectively, and these yielded FDR-corrected significant predictor variables of blood HbA1c (3.51), blood vitamin D (−2.91), salivary α-glucose (−2.85), salivary lactate (2.16), salivary taurine (1.84), salivary creatinine (1.78), and a salivary ISB admixture consisting of histidine, phenylalanine and dimethylsulphone resonances (1.76), in that order; significant d values are provided in brackets, with positive and negative values representing those which had higher concentrations in the T2DM and ND groups, respectively. However, corresponding (z) values for the EBAM analysis were significant only for blood HbA1c (3.51), blood vitamin D (−2.91), and salivary α-glucose (−2.85). 

Subsequent to the selection of these FDR-corrected predictor variables through the application of these strategies, a repeated AUROC analysis was conducted using the SAM-selected biomarkers only (Figure 6c). This approach gave AUROC values ranging from 0.788 (2 variables only) to 0.864 (7 variables in total), i.e., it provided a stronger predictive model than that obtained with all 60 potential predictor variables incorporated (Figure 4). With the exception of the model containing only three discriminatory biomarkers, all AUROC values had 95% CI values with lower bounds being above the null hypothesis value of 0.50, and so these were all highly statistically significant, most notably because they had already been subjected to FDR correction via SAM analysis. 

The overall maximum predictive accuracy for the best model with only four biomarker variables was 82.8%, and this correctly predicted the disease group membership of 23/27 ND and 24/29 T2DM study participants. The significantly increased predictive accuracy value obtained for this FDR-corrected dataset over that of the uncorrected model demonstrated the merit of using this prior SAM-based approach. The rank frequency (RF) values of the seven variables employed for testing were α-glucose ≈ HbA1c ≈ lactate (all RF 1.00) > taurine (RF 0.36) > vitamin D (RF 0.34) > the admixed histidine/phenylalanine/dimethylsulphone ISB (RF 0.18) > creatinine (RF 0.12). The univariate AUROC values for those predictor variables were 0.552 for taurine, 0.520 for the histidine/phenylalanine/dimethylsulphone admixture, and 0.563 for creatinine. 

## 4. Discussion

**Figure 7:**
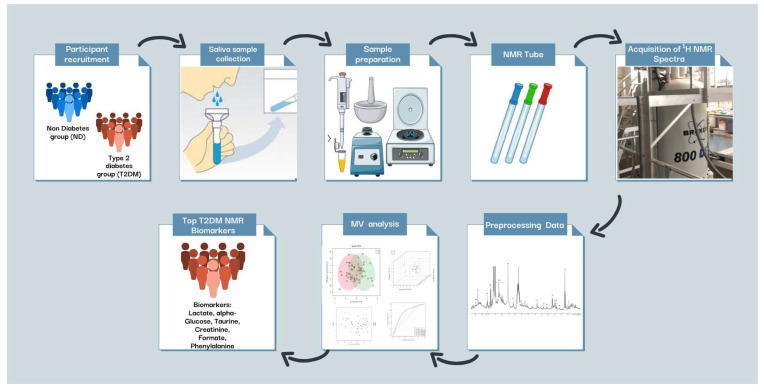
A schematic representation of the investigation conducted, showing the discovery of ^1^H NMR-detectable salivary biomarkers for T2DM and T2DM-linked dental caries.

The current study employed NMR-based metabolomics technologies to ascertain any differences in the human salivary metabolome arising from the induction and development of dental caries in patients with T2DM. Indeed, oral diseases such as dental caries and periodontitis have well-known associations (i.e., co-morbidities) with quite a substantial range of systemic disorders such as cardiovascular diseases and cancers, for example [2,3]. In addition to ^1^H NMR-detectable metabolites, this study also featured whole-blood HbA1c and 25-hydroxyvitamin D as co-marker species. A series of MV metabolomics techniques were deployed to explore differences between the patterns of biomolecules detectable in stimulated saliva samples collected from T2DM patients, and corresponding age-matched healthy controls. The results derived from a SAM-supported (FDR-adjusted) AUROC analysis demonstrated that α-glucose, HbA1c, lactate, taurine, 25-hydroxyvitamin D, and creatinine served as key biomarkers for differences between the salivary metabolite pools of the two groups investigated. Further discriminatory biomarkers were identified from the application of the PLS-DA and OPLS-DA strategies, and these included formate, phenylalanine, taurine, and urea, amongst others. An experimental schematic investigation chart which encompasses the study performed and the results acquired therefrom is shown in Figure 7.

The advantages of employing human saliva over other biospecimens for metabolomics studies include its simplicity of collection and a minimum level of invasiveness for patients who might be uncomfortable having their blood drawn, and also viability at study locations where the collection of such ‘invasive’ samples are neither safe nor practicable [25]. Salivary glands, oral mucosa, and gingival crevicular fluid (GCF), as well as the upper respiratory tract and possibly gastrointestinal system, release a variety of proteins and metabolites to saliva [26]. Moreover, human saliva contains transcriptomes and proteomes, and human metabolome measurements have been conducted previously to distinguish healthy individuals from diseased subjects [13,14,16,18,27,28,29,30].

In current metabolomics studies, both mass spectrometry and NMR spectroscopy are two analytical techniques that serve to detect and quantitate the concentrations of various metabolites in human or animal model biofluids [31]. NMR spectra yield much valuable information regarding the concentrations and physicochemical conditions of salivary metabolites, the latter including both their protein- and non-protein-bound forms. The main advantages of NMR spectroscopy include its minimal sample handling, the unbiased quantification of low-molecular-mass compounds in saliva, and high reproducibility [16,18,32,33]. However, using ^1^H NMR spectroscopy for salivary metabolomics studies is less common when compared to those performed on blood plasma and urine. In addition, preliminary studies using this technique have revealed metabolic modifications associated with many oral and systemic metabolic disorders [34]. 

Diabetes type 2 is a major metabolic disorder recognised as a risk factor for sub-optimal oral health, with implications for periodontal disease, xerostomia, and dental caries [35,36]. Previous studies conducted have shown an association of T2DM with oral diseases, such as periodontal disease [37] and tooth loss [38]. The impairment in salivary metabolites in dental research has been previously found in patients with dental caries. Indeed, in one NMR-linked metabolomics study, Fidalgo et al. [39] showed high levels of short-chain fatty acids in the saliva of children with dental caries. Another NMR study proposed a salivary metabolic biomarker profile consisting of formate, lactate, proline, and glycine in order to characterise early childhood caries [40]. However, investigations exploring the metabolite profiles of patients with T2DM and its association with oral health conditions are currently limited. 

In 2017, the joint European Federation of Periodontology/European Organisation for Caries Research (EFP/ORCA) working group provided a report focused on reviewing ecological interactions of dental biofilms in both health and disease, the involvement of microbial populations in the pathogenesis of dental caries and periodontal diseases, and the host responses to these disorders [41]. These reviewers found and concluded that these oral diseases were linked to a health-linked biofilm comprising *Veillonella, Granulicatella. Neisseria, Streptococcus*, and *Actinomyces* microorganisms, along with further genera; these microorganisms are organised as multispecies biofilms, which are metabolically specialised in the oral environment. Notably, the progression of periodontitis and dental caries features a wide range of microbial interactions induced by various stress factors. Indeed, in dental caries, the exposure of dental biofilms to dietary carbohydrates, and short-chain carboxylic acids derived from their microbial catabolism, enhances the generation of acidogenic and aciduric microbes.

Furthermore, more recently, Moussa et al. [42] reported on the current status and challenges presented by the global outcomes of dental caries research in the modern ‘omics’ era. Indeed, they presented a consensual conclusion arising from a wide range of microbiome investigations involving biologically genomic datasets, along with those regarding the metabolic end-products of these species in the oral environment, which stated that dental caries represents a community-scale metabolic disease with an aetiology involving much more than just a single causative organism. Such a consensus considered the important roles of newly developed sequencing techniques and omics technologies, i.e., metagenomics and metatranscriptomics, to inform researchers on the community constitution of the oral microbiome and its utilitarian activities in vivo. These researchers also noted the high value offered by evolving metabolomics and proteomics technologies, the former including high-resolution NMR analysis as in the current study, towards the provision of quantitative translational results. 

Moreover, in 2022, this research group presented a very novel development, specifically the very first ex vivo model of the early onset of dental caries with integrated multiomics [43]. This model centred on determinations of disease aetiologies at a clinically undiagnosable phase, and a longitudinal ex vivo system, involving a bioimaging protocol featuring non-invasive multiphoton second harmonic production, was employed in order to seek and monitor the very early signs of this disorder in human teeth employing supragingival plaque microcosms. Intriguingly, this model allowed for the identification of the exact disease onset, together with a synchronous delineation of modifications to the microbiome with metagenomics and targeted metabolomics techniques, and also explored co-occurrences between the microbial and metabolic biomarkers found. Key biomarkers identified were upregulated pyruvate, lactate, dihydroxyacetone phosphate, glyceraldehyde 3-phoshate, and downregulated fumarate, which all sequentially corresponded to selected bacterial taxa irrespective of their abundance. This observation provided evidence for the significance of bacterial activity over taxonomic abundance with regard to the mediation of pathogenic caries developments. The earlier biomarkers detected were viewed to be of critical importance for our understanding of the mechanism and dynamics of this disease process, especially with regard to the design and development of therapeutic interventional regimens and their timely application. Although all these biomarkers are readily ^1^H NMR-detectable and quantifiable (and the phosphate metabolites also by ^31^P NMR analysis), we commonly detect lactate, pyruvate, and sometimes fumarate in the ^1^H NMR profiles of WMSS specimens investigated. In the current study, lactate was found to be significantly upregulated in the caries-prone T2DM group, and this observation is consistent with the results found in ref. [43]. 

In the current study, ^1^H NMR-based salivary metabolomics was employed to explore salivary metabolic patterns underlying disease pathophysiology in T2DM patients with dental caries. These studies featured high-resolution ^1^H NMR spectroscopy, coupled with the orthogonal projections to latent structures discriminant analysis (OPLS-DA) and partial least squares discriminatory analysis (PLS-DA) models, amongst others. PLS-DA and OPLS-DA are both supervised statistical techniques that determine the best difference between phenotypic groups evaluated, as opposed to the unsupervised principal component analysis (PCA). Indeed, both these approaches are helpful when working with datasets that contain many significantly correlated variables, such as metabolite concentrations, since they allow for enhanced biomarker identification by distinguishing between such groups [44]. Notably, OPLS-DA views all variables as having some degree of inter-dependency. In PCA and PLS-DA, however, components (PCs) are formed as linear combinations of independent variables (metabolite levels), and all PCs are orthogonal, i.e., they are uncorrelated. The PLS-DA and OPLS-DA techniques generate a variable importance plot (VIP) value for each metabolite monitored, and this represents the significance of key variables in distinguishing between the groups assessed. In the current study, the results acquired from the application of both the PLS-DA and OPLS-DA models suggested that at least several metabolites in saliva differed significantly between the two groups compared herein.

Additionally, OPLS-DA represents an MV analysis approach that features the resolution and segregation of a continuous explanatory variable x*_i_* dataset into two factors, which can then be utilised to determine important output variables, e.g., a disease or stratified disease severity grading classification. The first of these comprises predictive data, whilst the second consists of uncorrelated, unpredictable information. This strategy generates models with an amplified level of diagnostics in metabolomics/biomarker research studies, together with more comprehensible visualisation options for these contributions. Nevertheless, this approach only enhances the interpretabilities, and not the predictivities, of supervised PLS techniques. 

The above PLS techniques discovered that lactate, α-glucose, taurine, creatinine, HbA1c, and phenylalanine, and to a lesser extent methanol and formate, were all significantly elevated in individuals with T2DM when compared to the ND group, whereas 25-hydroxyvitamin D and the Glyc A ^1^H NMR glycoprotein signal were downregulated in individuals with this disease. Urea and proline were also downregulated in this disease group, but this was found to be the case for only selected models applied. For example, downregulated proline, lysine, histidine, and tyrosine were only detected as significant distinguishing markers with the application of the OPLS-DA approach. 

Lactate and formate served as two of the key differentiating biomarkers, and their salivary concentrations were significantly elevated in the T2DM group over that of the ND classification. This finding was consistent with previous studies [45,46], although in blood serum, enhanced lactate concentrations are likely to arise from diabetic ketoacidosis [47]. These bacterial catabolites are produced by various species of bacteria, which can cause enamel demineralisation through a reduction in the pH value of the oral environment. Indeed, the acid form of lactate (lactic acid), in particular, has been previously identified as a major contributor to the initiation and progression of dental caries. Moreover, earlier investigations have found a notable link between lactic acid generated by *S. mutans* and the emergence of dental caries, and a positive correlation has been observed between the quantity of it produced by this bacterium and this disorder’s severity status [48,49].

*Actinomyces naeslundii* has been linked to the maintenance of a healthy balance between oral biofilm status and commonly found healthy microbiota [50,51]. Indeed, this species is known to raise local pH levels by producing ammonia and alkali [52,53], and can even transform lactic acid into a weaker acid, specifically acetate/acetic acid via an oxidative decarboxylation process involving pyruvate [54]. However, with frequent acid challenges, *A. naeslundii* can shift towards a pathogenic phenotype and further contribute to the development of dental caries [55,56]. In this respect, Shu et al. [57] reported that *A. naeslundii*, which is known to be a significant producer of formic acid, was positively associated with the development of dental caries. Carlsson [58] indicated that a combination of lactic and formic acids produced by *S. mutans* and *A. naeslundii*, respectively, can cause enamel demineralisation and contribute to the pathogenesis of dental caries, respectively. However, the role of this species in initiating dental caries is still not well understood [59,60].

Another significantly upregulated metabolite in T2DM is taurine, a sulphonate group-containing β-amino acid. Taurine has a crucial function in the cellular response to osmotic stress since this metabolite regulates changes in volume and the composition of saliva by modulating sodium flux [61]. However, interestingly, the reverse effect was observed in the current literature, although the measurements reported in this communication were made on human blood serum and not saliva; these researchers found that serum taurine was downregulated in diabetes, and from this observation, they concluded that this observation arose since this metabolite plays a crucial role in glucose homeostasis [62,63]. In addition, taurine plays a role in bile acid conjugation by reducing inflammation and maintaining calcium homeostasis. It has also been reported to possess a protective effect against acute cardiovascular events [64,65]. Notably, this β-amino acid has the ability to prevent inflammation and impede injuries mediated by oxidative stress [66]. Recently, Stacy et al. [67] reported that taurine possesses useful antibacterial properties. However, to date, there remains a lack of evidence on the effects of taurine on cariogenic bacteria. 

Similarly, creatinine was observed to be elevated in the T2DM group, which was consistent with previous studies of high blood serum creatinine levels being observed in patients with diabetes [68]. Diabetes can affect the glomerular filtration system by reducing the capability to filter metabolic waste products from the blood, and therefore cause creatinine accumulation in the circulation [69].

Additional significant findings based solely on the PLS-DA model were that the salivary concentrations of phenylalanine and methanol were also found to be elevated in T2DM (with VIP values marginally greater than one). Phenylalanine in saliva has recently been reported to be positively correlated with periodontitis [70,71], which is linked to T2DM. In addition, a seven-year follow-up study conducted on Finnish men identified that specific amino acids, including phenylalanine, were correlated with elevated glucose levels [72], although this study again did not involve the analysis of human saliva specimens. Methanol has previously been reported to be highly correlated with periodontitis [73] and was also detected at high levels in exhaled breath analysis using gas chromatography [74]. However, this alcohol may arise from tobacco cigarette smoking [14], and therefore its role as a T2DM biomarker remains limited. 

Interestingly, in the current study, salivary α-glucose was reported to be downregulated in patients with diabetes, an observation which requires further investigation since most studies have reported a positive correlation between salivary and blood glucose levels [75,76]. However, any such correlations will be critically dependent on the rate of transfer of this metabolite from the host blood source weighed against that of its consumption by the bacterial load within the oral environment, both of which will be expected to be upregulated in T2DM patients over those of age-matched control participants. Our previous investigations have shown that salivary metabolites may be segregated into four clear principal components, of which the two most important (i.e., those with the two highest levels of total model variance accountable) were those derived from host and microbial sources [14]. Nevertheless, other studies have shown that diabetics had higher levels of salivary glucose in patients with periodontal diseases [77] and dental caries [78]. 

Another significantly downregulated entity in both models was the Glyc A glycoprotein resonance. The species responsible for this signal originates from a subset of N-acetylglucosamine (GlcNAc) and terminal N-acetylneuraminate (silate) residues on glycosylated ‘acute-phase’ N-acetylated glycoproteins, which are characterised by a composite broad signal located at or close to 2.04 ppm [79]. However, a previous study reported a positive correlation between the Glyc A signal and insulin resistance in serum or plasma [80]. Urea was also observed as a significantly downregulated metabolite, albeit only in the PLS-DA model, which also requires further assessment since existing reports suggested that urea levels in the blood are putatively positively correlated with glucose and HbA1c levels in this biofluid [81]. In addition, a comparative study related to saliva samples collected from individuals with diabetes and xerostomia reported similar associations [82]. Finally, a systematic review and meta-analysis conducted reported serum proline levels to be higher in patients with diabetes [83]. 

Lysine and tyrosine were both significantly downregulated in the OPLS-DA model applied here. Both trends appeared to be consistent with other NMR studies exploring blood serum samples collected from individuals with diabetes [83,84]. It should also be noted that increased lysine contents were reported in the parotid saliva of caries-free individuals [85]. The cariostatic effect of lysine has been studied in both animal and human models. In the former studies, lysine was shown to reduce the incidence of caries in rats and hamsters when added to their diet [86]. In the latter investigations, however, the effect of this amino acid on caries prevention was investigated both topically and systemically. Notably, topical application using a poly-lysine-containing toothpaste has been shown to reduce the numbers of *Streptococcus mutans* [87] in the oral environment. In this respect, systemic administration of lysine supplements has also been investigated, with a few studies suggesting potential benefits in preventing caries development [88,89]. The mechanism by which lysine exerts its cariostatic effect is not fully understood; however, it may be speculated that its ability to interfere with the adherence and growth of cariogenic bacteria, as well as its role in collagen biosynthesis and the repair of oral tissues, could indeed be the contributory factors for this therapeutic effect [90]. 

One limitation of this study was the use of paraffin as a salivary stimulant, which generated quite intense polyol signals in the carbohydrate region, i.e., signals ascribable to D-glucitol, D-xylitol, and D-mannitol, as well as enzactin, stearate, and glycerol fatty acid ester (GFAE) species. However, these resonances arising from exogenous compounds were successfully removed from the spectra acquired prior to metabolomics analysis. Therefore, future studies should use physiologically relevant methods for saliva collection, such as the passive drool collection regimen in order to avoid such complications. 

Of especial interest to this stimulatory approach to saliva sample collection, in 2004, Gavião and Van der Bilt [91] investigated the rate of saliva secretion in 16 healthy participants while chewing natural and artificial foods, together with Parafilm wax, with the latter representing a malleable, ductile, odourless, semi-transparent, self-sealing thermoplastic stimulatory material consisting of a blend of waxes and olefines (of course, it is also odourless and non-toxic). These studies revealed that the flow rates determined both with and without Parafilm stimulation were markedly significantly lower than those observed with food chewing episodes. However, salivary flow rates observed with and without stimulation, including that with Parafilm, and that with chewing on a variety of foods tested, were all correlated significantly.

However, how does stimulation with paraffin wax or Parafilm affect the determination of salivary agents, including biomolecules and biomarkers? As noted above, efficient sample collection may be problematic in view of the ability of stimulants to interfere with valid biomolecular determinations, via ^1^H NMR analysis or otherwise. Notably, in 2013 Neyraud et al. [92] investigated the influence of stimulating saliva prior to its collection with parafilm laboratory film for a 5 min. duration and compared results acquired from a non-stimulated control group (saliva was collected via study participants permitting its free flow for 5 min. into pre-weighed vessels). ^1^H NMR analysis followed by the application of the PLS-DA segregation technique demonstrated that a number of amino acids and organic acid anions, along with fatty acids, were upregulated in the simulated collection group; it appeared that the amino acids arose from the degradation of proline-rich proteins. However, both taurine and propionate were found to be significantly underregulated in this group. The authors therefore concluded that the careful control of saliva sampling strategies is required, or at least must be considered for metabolomics investigations featuring the monitoring of biomarkers. Hence, any modifications to such metabolites noted in our study, particularly those to proline and taurine (down- and upregulated in the T2D group, respectively (Table 3)), could be partly confounded by the use of the paraffin wax stimulatory material for WMS sample collection in the current study. Saliva is a biological fluid that is easy to collect and is of considerable interest as a source of biomarkers. To date, its protein composition has been the most extensively studied but its metabolic composition is also of real interest. However, the composition of saliva is complex and dependent on numerous factors, among which stimulation is a source of many variations. The aim of this work was to study the effects of a stimulating condition (chewing) versus a resting condition on the human salivary metabolome. Saliva from 16 subjects was collected on three occasions and studied using nuclear magnetic resonance. The two conditions could be separated by PLS-DA analysis. Fatty acids, some organic acids, and amino acids, probably arising from the degradation of proline-rich proteins, were over-represented in stimulated saliva, whereas taurine and propionate were over-represented in resting saliva. To clarify further the identification of fatty acids, the free and total fatty acid contents were studied by gas chromatography. The principal fatty acids identified were oleic, stearic, and palmitic acids. It was also possible to separate the two conditions of stimulation by PLS-DA. These results show that the regulation of saliva and sampling conditions must be taken into account when studying markers in saliva.

Furthermore, Dlugash and Schultheiss [93] explored the influence of the use of salivary stimulants on analytical measurements obtained on steroid hormones present in this biofluid using a radioimmunoassay technique, including commonly determined cortisol and testosterone. From this investigation, it was found that there were no significant differences between the concentrations of these agents in saliva samples which were unstimulated and Parafilm-stimulated, and therefore the authors concluded that this flow stimulant was acceptable for assaying these hormones in this biofluid. 

Hence, despite the many complications associated with the use of paraffin wax as a salivary flow stimulant, notably the release of many potentially interfering ^1^H NMR-detectable exogenous agents therein into the saliva samples collected for analysis, it would appear that the stimulatory process employed in the current study may not significantly impact the determinations of salivary biomolecules using this technique. This study also demonstrated that this interfering issue may be successfully circumvented via the complete removal of ^1^H NMR resonances arising from these interferants from the salivary spectral profiles acquired prior to metabolomics analysis, although this process was found to be rather onerous and time-consuming, and obviously not all endogenous salivary ^1^H NMR bucket variables could be included in the metabolomics analysis conducted. Nevertheless, further investigations may be required, including those focussed on the ability of this salivary stimulant to influence salivary flow rates, in order to explore such effects further.

## 5. Conclusions

In conclusion, this study successfully identified a series of salivary metabolites that differ significantly between individuals with T2DM and healthy controls. These results provide important insights into the metabolic changes that occur in saliva in individuals with T2DM and their potential role in facilitating the development of dental caries. These promising biomarkers, which were predominantly verified by FDR-adjusted SAM analysis prior to the application of the MV AUROC strategy, included T2DM-upregulated salivary lactate, taurine, creatinine, formate, and downregulated salivary α-glucose and blood vitamin D, along with upregulated blood HbA1c. Interestingly, both lactate and formate have been previously implicated as significant biomarkers for dental caries [39,40,54]. Therefore, it is suggested that a more extensive study of this nature is performed with larger numbers of participants, if indeed feasible. 

The identification of salivary biomarkers for diabetes and its associated complications could have significant clinical implications, particularly in improving the accuracy of diagnosis and the monitoring of the condition, along with the provision of valuable information concerning their roles in predicting T2DM-induced oral diseases such as periodontal diseases and dental caries. Notably, the detectable biomarkers are conceivably of much importance for enhancing our understanding of the microbiomics- and/or metabolomics-based pathogenesis of both periodontal diseases and dental caries and may facilitate the therapeutic targeting of specific microbial species that play key roles in the pathogenic development and progression of these disease processes. They may also serve to aid the design, development, and characterisation of new therapeutic approaches for the treatment, prophylactic or otherwise, of these oral conditions, together with the timing of these measures, most especially during their early stages. 

## Figures and Tables

**Table 1:** Clinical characteristics for the ND and T2D groups involved in this investigation (mean ± SD blood HbA1c levels and ICDAS dental caries severity indices, along with the % distribution of root caries lesions, and the % extreme risk of dental caries).

Clinical Characteristic	ND	T2DM	*p* Value
Blood HbA1c concentration (mean ± SD)	35.70 ± 5.94 mmol/mol	54.15 ± 13.03 mmol/mol	1.11 × 10^−8^
Severity of dental caries: mean of ICDAS ± SD	0.57 ± 0.24	0.67 ± 0.32	0.014
Distribution of root carious lesions (%)	21%	76.9%	na
Extreme risk of dental caries (%)	48.9%	16.48%	na

**Table 2:** Assignments of resonances in the 800 MHz ^1^H NMR spectra of supernatants of SWS samples obtained from the ND group (chemical shift (δ) values and resonance coupling patterns are also provided). Assignments and assignment codes represent those from refs. [14,16,18,20]. Abbreviations: *s*, singlet; *d*, doublet; *t*, triplet; *q*, quartet; *m*, multiplet.

Assignments	Assignment Code	Chemical Shift (δ/ppm)	Coupling Pattern
Leucine-CH_3_	7	0.962	*t*
Propioniate-CH_3_	11	1.058	*t*
*Iso*-Butyrate-CH_3_	12	1.125	*t*
Ethanol-CH_3_	15	1.183	*d*
Methylmalonate-CH_3_/α-L-Fucose-CH_3_	16	1.211	*d/d*
3-D-hydroxybutyrate-CH_3_/β-Fucose-CH_3_	17	1.242	*d*
Lactate-CH_3_	18	1.330	*d*
Acetoin-CH_3_	19	1.371	*d*
Alanine-CH_3_	20	1.486	*d*
5-Aminovalerate-β,γ-CH_2_′ s	22	1.641	*m*
Leucine-CH_2_	23	1.685	*m*
Senicioate-CH_3_	27	1.823	*s*
Thymine-CH_3_	Uncoded	1.860	*s*
Acetate-CH_3_	30	1.920	*s*
2-Hydroxyglutarate-γ-CH_2_	31	1.954	*m*
Proline-γ-CH_2_/N-Acetylneuraminate-C3H	32	2.005	*m*
Glycoprotein carbohydrate side-chain N-acetylsugar-NHCOCH3 groups (Glyc A sugnal)	33	2.020–2.080 (3 partially resolved signals)	*broad*
N-Acetylglutamate-/N-Acetylaspartate–NHCOCH_3_ (2 signals)	34	2.025/2.030	*s*
N-Acetylglucosamine-/N-Acetylneuraminate-NHCOCH_3_	35	2.040/2.060	*s*
Unassigned N-Acetylated metabolite-NHCOCH_3_ (2 signals), including N-Acetylneuraminate (2.060 ppm)	36	2.053/2.060	*2 × s*
Propioniate-CH_2_	40	2.193	*q*
Acetone-CO-CH_3_	41	2.215	*s*
5-Aminovalerate-α-CH_2_	42	2.235	*t*
Pyruvate-CH_3_	44	2.377	*s*
Succinate-CH_2_	46	2.415	*s*
Dimethylamine-N(CH_3_)_2_	48	2.723	*s*
Trimethylamine-N(CH_3_)_3_	49	2.872	*s*
5-Aminovalerate-δ-CH_2_/Lysine-ε-CH_2_	50	3.004	*t/t*
Creatine-N(CH_3_)	51	3.022	*s*
Dimethylsulphone-CH_3_ (3.10 ppm)/1/2 His- and Phe-β-CH_2_ (3.14 ppm)	54	3.10–3.15	*s/m*
1,9-Dimethylurate-N1(CH_3_)	55	3.183	*s*
Betaine-N(CH_3_)_3_^+^/Taurine-CH_2_NH_3_^+^	58	3.242	*s/t*
Paraxanthine-N(CH_3_)	61	3.328	*s*
1,3,7-Trimethylurate-N7(CH_3_)	62	3.348	*s*
Methanol-CH_3_/1,3-Dimethyluracil-N1(CH_3_)	64	3.386	*s/s*
Urea-CO-NH_2_	98	5.790	*broad signals*
Uracil-C2H	99	5.800	*d*
Protein aromatic amino acid residue(s)	102	6.850	*broad*
Tyrosine aromatic ring-C2H/C6H	103	6.880	*d*
Histidine imidazole ring-C5H	104	7.071	*s*
Hydroxyphenylacetate-aromatic ring-C2H/C6H	Uncoded	7.17	*d*
Tyrosine aromatic ring-C3H/C5H	105	7.237	*d*
Phenylalanine aromatic ring-C2H/C6H	106	7.320	*m*
Phenylalanine aromatic ring-C4H	107	7.375	*m*
Phenylalanine aromatic ring-C3H/C5H	108	7.430	*m*
Uracil-C1H	109	7.533	*d*
Protein aromatic amino acid residue(s)	110	7.552	*m*
Histidine imidazole ring-C2H	111	7.812	*s*
Protein aromatic amino acid residue(s)	113	8.050	*2 × broad signals*
Hypoxanthine-C8H	114	8.175	*s*
Hypoxanthine-C3H	115	8.219	*s*
Formate-H	116	8.456	*s*

**Table 3:** Decoding metabolite signatures: Insights from the PLS-DA model on chemical shift markers, their assignments [14,16,18,20], variable importance parameter (VIP) values, and regulatory status. * The up arrow indicates that metabolites were upregulated with higher levels in the T2DM WMS samples, whereas the down arrow indicates those which were downregulated in this group (i.e., their values were higher in the ND group).

Chemical Shift (ppm)	Corresponding Assignment	VIP Value	Regulatory Status *
3.40–3.46	Taurine-CH_2_SO_3_^−^	3.14	↑
3.03–3.08	Creatinine-NCH_3_	2.92	↑
3.10–3.15	½ Histidine-/Phenylalanine-β-CH_2_/Dimethylsulphone-SO_2_(CH_3_)_2_	2.91	↑
5.24–5.28	α-Glucose-C1H	1.88	↓
HbA1c	n/a	1.78	↑
1.30–1.33 ppm	Lactate-CH_3_	1.75	↑
Vitamin D	n/a	1.49	↓
3.27–3.30 ppm	Unassigned	1.39	↑
2.00–2.06 ppm	Glyc A glycoprotein-NHCOCH_3_	1.17	↓
5.74–5.80	Urea-CONH_2_	1.17	↓
7.39–7.43	Phenylalanine aromatic ring-C3H/C5H	1.02	↑
4.13–4.19	Lactate-CH	1.02	↑
3.30–3.36	Methanol-CH_3_	1.01	↑
8.41–8.47	Formate-H	0.90	↑

**Table 4:** Decoding metabolite signatures: Insights from the OPLS-DA model on chemical shift markers, their assignments [14,16,18,21], variable importance parameter (VIP) values, and regulatory status. * For the latter, up and down arrows indicate higher and lower salivary concentrations, respectively, in the T2DM group.

Chemical Shifts (ppm)	Corresponding Assignment	VIP Value	Regulatory Status *
HbA1c	n/a	3.44	↑
Vitamin D	n/a	3.38	↓
1.30–1.33 ppm	Lactate-CH_3_	2.06	↑
2.00–2.06 ppm	Glyc A glycoprotein-NHCOCH_3_	1.69	↓
4.13–4.19 ppm	Lactate-CH	1.43	↑
1.95–1.97 ppm	Proline-γ-CH_2_	1.32	↓
1.66–1.72 ppm	Lysine-CH_2_	1.29	↓
3.40–3.46 ppm	Taurine-CH_2_SO_3_^−^	1.28	↑
1.97–2.00 ppm	Proline-β-CH_2_	1.27	↓
7.77–7.82 ppm	Histidine imidazole ring-CH	1.19	↓
3.10–3.15 ppm	½ Histidine-/Phenylalanine-β-CH_2_/Dimethylsulphone-SO_2_(CH_3_)_2_	1.16	↑
3.03–3.08 ppm	Creatinine-NCH_3_	1.11	↑
6.87–6.93 ppm	Tyrosine aromatic ring-C3/C5H	1.01	↓

**Table 5:** Important variables selected from AUROC analysis, including ^1^H NMR bucket assignments [14,16,18,21], their regulatory status, and computed univariate AUROC values. This dataset was constant sum-normalised (CSN), glog-transformed, and Pareto-scaled prior to analysis. * The up arrow indicates metabolites upregulated (higher levels) in the T2DM group, whereas the down arrow indicates those downregulated in the T2DM group (i.e., those higher in the ND group).

Variable or ^1^H NMR Bucket	^1^H NMR Assignment	Regulatory Status *	Univariate AUROC Value
HbA1c	n/a	↑	0.736
Vitamin D	n/a	↓	0.727
5.24–5.28 ppm	α-Glucose-C1H	↓	0.700
1.30–1.34 ppm	Lactate-CH_3_	↑	0.683
5.72–5.74 ppm	Urea-CONH_2_	↓	0.625
5.74–5.76 ppm	Urea-CONH_2_	↓	0.617
7.39–7.43 ppm	Phenylalanine-C3H/C5H	↑	0.611
5.08–5.14 ppm	Unassigned	↑	0.567
8.41–8.47 ppm	Formate-H	↑	0.582
4.13–4.19 ppm	Lactate-CH	↑	0.571
7.04–7.09 ppm	Histidine imidazole ring-C5H	↓	0.531
7.99–8.01 ppm	Unassigned	↑	0.531
7.35–7.39 ppm	Phenylalanine aromatic ring-C3H/C5H	↑	0.610
4.11–4.13 ppm	Unassigned	↓	0.521
7.12–7.17 ppm	4-Hydroxyphenylacetate aromatic ring-C2H/C6H	↑	0.554
5.19–5.24 ppm	Unassigned (apparent triplet)	↓	0.548
1.01–1.07 ppm	Valine-CH_3_	↑	0.525
3.30–3.36 ppm	Methanol-CH_3_	↑	0.581
7.30–7.35 ppm	Phenylalanine aromatic ring-C4H	↑	0.521

## Data Availability

The data presented in this study are available in this article.

## Supplementary Materials

The following supporting information can be downloaded at: https://www.mdpi.com/article/10.3390/metabo14070372/s1, Table S1. Participants’ Criteria.
