# Supplementary material for: Exploring Salivary Metabolic Alterations in Type 2 Diabetes: Implications for Dental Caries and Potential Influences of HbA1c and Vitamin D Levels
Source: Metabolites. 2024 Jun 30;14(7):372. doi: 10.3390/metabo14070372 (PMC11279097; doi:10.3390/metabo14070372)
Supplement: Supplementary file 1 [file metabolites-14-00372-s001.zip › metabolites-2957512-supplementary.pdf]

**Supplementary Table S1. Participants' Criteria**

| Inclusion Criteria | Exclusion Criteria |
|---|---|
| • Participants who are male or female ≥ 18 years of age.<br>• For the test group, they have been diagnosed with type 2 Diabetes.<br>• For the control group, participants who are not diagnosed with type 2 Diabetes.<br>• Participants having minimum of six natural tooth.<br>• They are capable of giving informed consent.<br>• They have the ability to understand and speak English.<br>• They are able and willing to comply with all trial requirements.<br>• They are not participating in another dental trial.<br>• They are not diagnosed for cognitive defect due to mental illness, depression, Alzheimer's disease, or dementia.<br>• No antibiotics, no steroidal and/or non-steroidal anti-inflammatory medication used during the last 3 weeks.<br>• Participants who are not pregnant and also not breastfeeding<br>• Participations who are not in another dental study testing different dental products during the previous three months and during the study period<br>• Participants who are not currently taking Vitamin D supplement | • Participants who are edentulous<br>• Cognitive defect due to mental illness, depression, Alzheimer's disease, or dementia.<br>• The presence of any hard or soft tissue tumours in the oral cavity<br>• Patients undergoing chemo and/or radiation therapy.<br>• Any other significant disease or disorder which, in the opinion of the Investigator, may either put the participants at risk because of participation in the trial, or may influence the result of the trial, or the participant's ability to participate in the trial.,<br>• Any condition, which in the opinion of the investigator, would preclude participation by the subject (such as cross-infection control risk)<br>• Participants who are prescribed long-term systematic antibiotics.<br>• Participants who are pregnant and breastfeeding<br>• Participations who are in another dental study testing different dental products during the previous three months and during the study period<br>• Participants who had additional fluoride treatment in the past 6/3 months<br>• Participants who are prescribed to use high fluoridated toothpaste.<br>• Participants who are currently taking Vitamin D supplements |
